# New Therapies for Highly Sensitized Patients on the Waiting List

**DOI:** 10.34067/KID.0000000000000509

**Published:** 2024-07-12

**Authors:** Ashley Vo, Noriko Ammerman, Stanley C. Jordan

**Affiliations:** Transplant Center, Cedars-Sinai Medical Center, West Hollywood, California

**Keywords:** acute rejection, chronic allograft rejection, clinical trial, ESKD, immunology and pathology, intravenous immunoglobulin, kidney transplantation, transplant outcomes

## Abstract

Exposure to HLA alloantigens through pregnancy, blood products, and previous transplantations induce powerful immunologic responses that create an immunologic barrier to successful transplantation. This is commonly detected through screening for HLA antibodies using Luminex beads coated with HLA antigens at transplant evaluation. Currently accepted approaches to desensitization include plasmapheresis/low-dose or high-dose intravenous Ig plus anti-CD20. However, these approaches are often unsuccessful because of the inability to remove high titer circulating HLA antibodies and limit rebound responses by long-lived anti-HLA antibody secreting plasma cells (PCs) and memory B cells (B_MEM_). This is especially significant for patients with a calculated panel reactive antibody of 99%–100%. Newer desensitization approaches, such as imlifidase (IgG endopeptidase), rapidly inactivate IgG molecules and create an antibody-free zone by cleaving IgG into F(ab’2) and Fc fragments, thus eliminating complement and cell-mediated injury to the graft. This represents an important advancement in desensitization. However, the efficacy of imlifidase is limited by pathogenic antibody rebound, increasing the potential for antibody-mediated rejection. Controlling antibody rebound requires new strategies that address the issues of antibody depletion and inhibition of B_MEM_ and PC responses. This will likely require a combination of agents that effectively and rapidly deplete pathogenic antibodies and prevent immune cell activation pathways responsible for antibody rebound. Here, using anti–IL-6 receptor (tocilizumab) or anti–IL-6 (clazakizumab) could offer long-term control of B_MEM_ and PC donor-specific HLA antibody responses. Agents aimed at eliminating long-lived PCs (anti-CD38 and anti–B-cell maturation antigen×CD3) are likely to benefit highly HLA sensitized patients. Complement inhibitors and novel agents aimed at inhibiting Fc neonatal receptor IgG recycling will be important in desensitization. Administering these agents alone or in combination will advance our ability to effectively desensitize patients and maintain durable suppression post-transplant. After many years of limited options, advanced therapeutics will likely improve efficacy of desensitization and improve access to kidney transplantation for highly HLA sensitized patients.

## Introduction

HLA sensitization poses a significant and substantial immunologic barrier to life-saving organ transplantation. Preformed HLA antibodies increase transplant list wait times, and if crossed without desensitization, it may result in increased risk for antibody-mediated rejection (AMR), graft failure, and patient mortality.^1–3^ The US Kidney Allocation System permits priority for sensitized candidates, allowing an increased opportunity for deceased donor kidney transplantation in HLA sensitized (HS) patients.^4,5^ Unfortunately, transplantation rates remain low for the most highly HS candidates (calculated panel reactive antibody [cPRA] >99.9%) because of unacceptable cross-matches.^6,7^ In fact, patients with this level of sensitization are more likely to die or be removed from the wait list than be transplanted.^6^ Several groups have reported promising results using desensitization for HLA incompatible kidney transplantation.^8–10^ Desensitization protocols have progressed considerably and have benefited from the introduction of therapies for autoimmunity and B-cell/plasma cell (PC) malignancies which decrease deleterious IgG molecules, target critical cytokines, and eliminate B cells and PCs. Another critical advancement has been our ability to stratify immunologic risk using Luminex single antigen bead assays.^11,12^ This allows a window of opportunity to successfully transplant HS patients with decreased risks. In this review, we will not discuss the basic concepts and approaches to desensitization. These basic concepts are discussed in references presented here.^6,8,13,14^ Many new desensitization strategies have emerged to address this vexing immunologic problem (Table 1). However, there are currently no US Food and Drug Administration agents approved for desensitization.

**Table 1:** Desensitization strategies

Drug/MOA	Reference	NCT#	Study Type	Published Information in Desensitization	Patients Enrolled	Results
IVIg Many formulations and manufacturers Inhibits B-cell and T-cell proliferation, upregulate anti-inflammatory Th2 cytokines, anti-idiotypic blockade of alloantibodies, enhance clearance of pathogenic IgG through blockade of FcRn	Jordan *et al.*, 2004	—	NIH IG02 RCT	High-dose: 2 g/kg monthly (max 140 g)×4 mo before and 4 mo after transplant^19^ or placebo	101 enrolled 98 analyzed	IVIg significantly reduced cPRA levels versus placebo. Sixteen IVIg patients were transplanted (35%) versus eight placebo (17%). Two-year graft survival rates were similar (80% IVIg, 75% placebo)
	Montgomery *et al.*, 2000	—	Retrospective review	Low-dose with PLEX: 100 mg/kg^92^	7	PLEX/IVIg allowed successful transplant without cases of hyperacute rejection Significant survival benefit for HS patients undergoing LDKT after desensitization with low dose IVIg and plasma exchange (PLEX) was shown in the subsequent Montgomery publication: the 8-yr survival of those receiving DES versus those who received HLA compatible KT or remained on hemodialysis 80.6%, 49.1%, 30.5%, respectively; *P* < 0.001^8^
**Anti-CD20**						
Rituximab (Rituxan) Genentech, San Francisco, CA (biosimilar products are available) mAb directed against CD20 antigen on the surface of B lymphocytes	Vo *et al.*, 2008	NCT00642655	Phase 1 Phase 2	1 g IV on days 7 and 22 with high-dose IVIg (2 g/kg on days 0 and 30)^93^	20	Mean waitlist time was reduced from 144±89 to 5±6 mo in 16 of 20 patients transplanted, with a 1-yr graft and patient survival of 94% and 100%, respectively
	Vo *et al.*, 2013	NCT01178216	Retrospective review	1 g IV on day 15 with high-dose IVIg (days 0 and 30)^94^	207	Transplantation occurred in 146 (71%) patients and at 48 mo, patient and graft survival were 95% and 87.5%, respectively
	Loupy *et al.*, 2010	—	Pilot study	375 mg/m^2^ with high-dose IVIg and PLEX^95^ Group 1 (*N* = 36) received four high-dose IVIg Courses, started before reperfusion, followed by days 21, 42, and 63 post-transplantation Group 2 (*N* = 18) received the same dose of IVIg, with rituximab+PLEX rituximab 375 mg/m^2^ at day 4, repeated depending on CD19 cells^95^	54, with preformed DSA and single ABO-compatible DDKT	At 12 mo post-transplant, group 2 had Lower microcirculation inflammation lesions (glomerulitis+capillaritis score of 1.8±0.2 versus 2.7±0.2, respectively, *P* = 0.03) Lower rate of transplant glomerulopathy (7% versus 38%, *P* = 0.02) Lower rate of cAMR (41.3% versus 13.3%, respectively, *P* = 0.03) Decline in DSA-MFI from day 0–1 yr was 44%±13% in group 1 compared with 80%±8% in group 2 (*P* = 0.02) eGFR was 43±16 versus 54±16 ml/min per 1.73 m^2^ in groups 1 and 2, respectively (*P* = 0.04)
	van den Hoogen *et al.*, 2015	NCT00565331	RCT	1:1 rituximab 375 mg/m^2^ versus NS IV ×1 during surgery^96^	280	The primary outcome, BPAR within 6 mo after transplantation, occurred in 23 of the 138 rituximab-treated patients (16.7%), compared with 30 of 142 placebo-treated patients (21.1%, *P* = 0.25)
Obinutuzumab Genentech Glycoengineered type 2 anti-CD20 mAb FDA approved for chronic lymphocytic leukemia	Redfield *et al.*, 2019	NCT02586051	Phase 1 (THEORY)	1 g IV single dose (*N* = 5) or two doses on days 1, 15 (*N* = 20) with high-dose IVIg followed by a dose at transplant and 24 wk post-transplant^33^	25	Peripheral and lymphoid B-cell depletion was noted but reductions in anti-HLA antibodies and cPRA score were not clinically meaningful for most patients. Eight patients were transplanted
**Anti-IL6/IL-6R**						
TCZ Genentech Recombinant humanized anti-human IL6 receptor mAb Binds both soluble and membrane bound IL-6R	Vo *et al.*, 2015	NCT01594424	Phase 1 Phase 2	8 mg/kg IV monthly (max per dose: 800 mg) IVIg (2 g/kg on days 1 and 30) and TCZ on day 15, then monthly for 6 mo up to transplant. Post-transplant, TCZ on day 2, then monthly for 6 mo^37^	10	Mean time to transplant from first desensitization of 25±10.5 mo decreased to 8.1±5.4 mo post-TCZ. Reduced strength and number of DSAs were seen at transplant (*P* = 0.024) and 12 mo post-transplantation (*P* = 0.0003). Five patients were transplanted and TCZ had an acceptable safety profile (primary endpoint)
	Daligault *et al.*, 2021	—	Single-arm, prospective pilot study	8 mg/kg IV monthly^38^	14	TCZ was able to significantly reduce dominant anti-HLA antibody sensitization. MFI decrease was minor compared with the initial values; this was insufficient to allow compatible KT, with only one patient transplanted
	Jouve *et al.*, 2023	—	Single-arm, prospective pilot study (TETRA)	Rituximab 375 mg/m^2^ ×2 doses; TCZ group received 8 mg/kg monthly ×6 doses before transplant^39^	33 (26 SOC and seven toci+SOC)	TCZ+SOC might be beneficial to reduce antibody rebound of high MFI antibodies, with longer lasting effects, but MFI reductions pretransplant were similar to SOC
Clazakizumab CSL Behring, King of Prussia, PA Genetically engineered, humanized IgG1 mAb; IL6 ligand inhibition	Vo *et al.*, 2022	NCT03380962	Phase 1 Phase 2, open label, single arm	25 mg subQ every 4 wk for 6 mo up to transplant, then q4 wks for 12 mo post-transplant, followed by a LTE study^41^	20	Eighteen patients were transplanted. DSA-positive patients at transplant showed reductions over 12 mo post-transplant, with only 1 DSA-positive at 1 yr. No patients developed dnDSA. AMR occurred in three patients (15%). Patient and graft survival were 100% and 94%, respectively, at 12 mo (one graft loss occurred because of surgical complications) Most patients showed significant increases in T_REG_ and B_REG_ cells at 6–12 mo post-transplant
	Not yet published	NCT03744910	Phase 3, RCT (IMAGINE)	12.5 mg subQ every 4 wk for approximately 5 yr for cABMR (average)^44^	350	Study was ongoing but closed early as of November 2023 because of a failure to show efficacy at 1 yr
**PC inhibitors**						
Bortezomib Takeda, Cambridge, MA First-generation reversible proteasome inhibitor	Eskandary *et al.*, 2018	NCT01873157	RCT, single center	1.3 mg/m^2^ IV on days 1, 4, 8, and 11 or placebo for AMR^45^	44	Failed to show that bortezomib prevents GFR loss, improves histologic or molecular disease features, with significant toxicity
	Moreno *et al.*, 2017	NCT00722722	Prospective, open-labeled, nonrandomized, trial	1.3 mg/m^2^ IV for 32 doses^46^	10	Only a modest reduction in DSAs, no change in cPRA. Most were not transplanted and/or experienced side effects of bortezomib
Carfilzomib Amgen, Thousand Oaks, CA Second-generation irreversible proteasome inhibitor	Tremblay *et al.*, 2019	NCT02442648	Pilot, phase 1 phase 2	20–36 mg/m^2^ IV for 12 doses followed by PLEX on days 47, 49, and 51^47^	13	MFI was modestly reduced in most, but antibodies rebounded to baseline levels in all patients within 5 mo post-desensitization
	Pham *et al.*, 2021	—	Retrospective case series report	20 mg/m^2^ over 10–30 min on days 1, 2, 8, 9, 15, and 16 for AMR in patients undergoing lung transplant. PLEX and IVIg were per SOC and not standardized^48^	28	Of 14 patients with class 1 dnDSAs, 11 (78.6%) had complete resolution of DSA. Of 28 patients with class 2 dnDSAs, 6 (21.4%) had complete resolution of DSA. Many C1q+patients (*n* = 17) became C1q− (*n* = 12) 16 (57.1%) patients died
Daratumumab Janssen, Raritan, NJ IgG1κ humanized mAb directed against CD38 (FDA breakthrough status in 2015)	Kwun *et al.*, 2019	—	Preclinical	16 mg/kg IV weekly for 4–8 doses^51^	Eight rhesus macaque monkeys	Significant reduction in DSA and slightly improved renal allograft survival. Rapid rebound of antibodies with profound CMR at 1 mo
	Jordan *et al.*, 2019	—	Case report	16 mg/kg IV weekly for 4–8 doses for DES in a heart transplant candidate and AMR treatment for HS KT patient^52^	2	Daratumumab effectively reduced HLA antibodies and improved ABMR. However, concerns for antibody rebound, B-reg depletion and CMR incitement may limit efficacy
	Vo *et al.*, 2023	—	Case series	1800 mg SQ weekly ×4 doses after failure of other DES therapies^55^	10	Daratumumab reduced DSA MFI strength and allowed for HLAi transplantation in eight of ten patients. Mean MFI at baseline observed for CI and CII were 7316±4286 and 11,179±3794, respectively, in transplanted patients. Two patients experienced rejection (AMR and mixed). At 12 mo, patient/graft survival was 100%/100% and eGFR was 73±22 ml/min per 1.73 m^2^. Daratumumab was well tolerated with no significant SAEs noted
Isatuximab Sanofi, Bridgewater, NJ IgG-derived mAb against CD38	Vincenti *et al.*	NCT04294459	Open-label single-arm phase 1/2 study	10 mg/kg weekly ×4; then Q2 weeks ×8 wk as DES monotherapy for patients with cPRA 80%–99.9% and ≥99.9%^97^	23	Study found overall cPRA values were minimally affected, with only 9/23 (39%) reached target cPRA reduction. Isatuximab was well tolerated with good safety profile, although risk of hypogammaglobulinemia was a concern
**Costimulatory blockade**						
Belatacept Bristol Myers Squibb, Princeton, NJ Selective T-cell blockade by binding CD80 and CD86 receptors on APC to block CD28 mediated costimulation and blocks the alternative ligand CTLA-4; high affinity CTLA4-Ig	Vincenti *et al.*, 2016	NCT00256750	(BENEFIT) Phase 3 RCT	More-intensive belatacept (0–3 mo: 10 mg/kg on days 1, 5 and weeks 2, 4, 6, 8, 10, 12 mo 4–6: 10 mg/kg at weeks 16, 20, 24 >6 mo: 5 mg/kg every 4 wk), less-intensive belatacept (months 0–1: 10 mg/kg on days 1, 5 and weeks 2, 4 months 2–3: 10 mg/kg at weeks 8 and 12 >3 mo: 5 mg/kg every 4 wk), or CSA^57^	660	Mean eGFR increased with both belatacept regimens but decreased with CSA (+1.30 per year bela versus −1.04 per year CSA)
**Combination therapy**						
Carfilzomib+belatacept second-generation irreversible proteasome inhibitor with costimulatory blockade	Jackson *et al.*, 2023	NCT05017545	Pilot Phase 1 Phase 2 (ADAPT)	20–27 mg/m^2^ for two to six doses with belatacept 10 mg/kg ×6 over 12 wk then 5 mg/kg QM ×10^61^	15 (prelim data reported *N* = 5)	Prelim data: A novel HLA DES regimen with PC depletion and costimulatory blockade was safe and reduced HLA antibodies; however, broad elimination of HLA antibodies was not yet realized and long-term data are needed. One patient received a transplant from a formerly compatible DDKT, with four historic DSAs, and is without rejection or DSA 4 mo post-transplant
Daratumumab+belatacept Anti-CD38 plus costimulatory blockade	Chandran *et al.*, 2023	NCT04827979	Phase 1 Phase 2 (ATTAIN)	Daratumumab 8 mg/kg weekly ×4 wk then every other week for 4 wk Belatacept 10 mg/kg q2 weeks starting at week 8 (weeks 8, 10, 12, 14)^60^	15 (prelim data reported *N* = 5)	Prelim data: A novel HLA DES regimen with PC depletion and costimulatory blockade appears safe and successful. In the initial subject, this regimen led to transplant without HLA antibody rebound or acute rejection. Long-term follow-up and more data are needed. AEs included acute cholecystitis and COVID (*N* = 1), upper GI bleed (*N* = 1) and fevers (*N* = 1); no opportunistic infection or malignancy were reported
Bortezomib+belatacept	Jain *et al.*, 2020	—	Retrospective report	Bortezomib plus belatacept^62^	6	Case reports of six patients with active AMR treated with bortezomib plus belatacept
**Anti-BCMA/CD3**						
REGN5459 (low affinity) and REGN5458 (high affinity) Regeneron, Tarrytown, NY Bispecific monoclonal targeting BCMA-CD3; proprietary human antibody mouse technology and full-length bispecific antibody platform	Not yet published	NCT05092347	Dose escalation phase 1, phase 2, open-label	REGN5459 IV×three weekly doses, in a dose escalation cohort; per protocol, if approved to proceed based on data in REGN5459, REGN5458 will open enrollment in a second dose escalation cohort^74^	60	Study is ongoing (now enrolling)
	Not yet published	NCT05106387	Follow-up observational study fir the dose escalation study	No additional study medication administered in the follow-up post-transplant study	Eligible patients from the feeder study	Study is ongoing (now enrolling). Patients who received at least one dose of REGN5459/5458 product from the feeder study and are transplanted are eligible to enroll in the follow-up study
**Imlifidase**						
Imlifidase (formerly IdeS) Hansa BioPharma, Lund Sweden Derived from *Streptococcus pyogenes* IgG endopeptidase that cleaves all four IgG antibodies into F(ab’)2 and Fc	Jordan *et al.*, 2017	NCT02426684	Phase 1 Phase 2 Open-label	0.24–0.5 mg/kg IV at transplant^65^ with alemtuzumab induction, IVIg and rituximab (US) or horse-derived anti-thymocyte globulin (Sweden)^65^	25	At transplant, IgG and HLA antibodies were eliminated. 24 patients were transplanted. Two graft losses were observed at 2.6 and 3.2 yr post-transplant (unrelated to imlifidase). Mean eGFR was 47.5 ml/min per 1.73 m^2^ at 3 yr
	Jordan *et al.*, 2020	NCT02790437	Phase 2, single arm (HighIdes)	0.25 mg/kg body weight IdeS on study day 0. If negative cross-match is not achieved, a second dose can be given within 2 d of the first infusion^64^	19	89.5% converted a baseline positive cross-match to negative within 24 h of imlifidase. Patient survival was 100% with graft survival of 88.9% at 6 mo
	Not yet published	NCT03897205	Phase 2	AMR: 0.25 mg/kg ×1 or 5–10 sessions of PLEX^98^	30	Study is completed but not yet published
	Not yet published	NCT04935177	Phase 3 (ConfIdeS)	0.25 mg/kg ×1 at transplant, followed by a possible second dose of 0.25 mg/kg within 24 h of dose 1^67^	64	Study is ongoing (now enrolling)
**Complement inhibition**						
Eculizumab Alexion, Boston, MA mAb binding protein C5, inhibiting cleavage to C5a and C5b and formation of MAC C5b-9	Stegall *et al.*, 2011 (Schinstock *et al.*,^6^ 2019—long-term follow-up)	NCT006707	Open label	1200 mg IV ×1 prior to transplant, 600 mg on POD1 then weekly ×4^78^	26	Significantly lower AMR incidence in the first 3 mo in HS recipients with eculizumab (*N* = 26; 7.7% eculizumab versus 41.2% historical controls) but no long-term improvement in allograft survival or cAMR rate over a mean follow-up of 6.8 yr
	Marks *et al.*, 2019	NCT01399593	Phase 2	Prevention of AMR: 1200 mg before transplant (day 0) eculizumab 900 mg (days 1, 7, 14, 21, 28), eculizumab 1200 mg (weeks 5, 7 and 9) versus SOC^99^	102	Terminated early for failure to achieve primary end point. Graft survival through month 36 was 91.8% for eculizumab and 78.5%, SOC *P* = 0.21
	Lefaucher *et al.*, 2018	Post hoc analyses of clinical trials: NCT01567085 and NCT01399593	Multicenter, international study; *post hoc* analyses	Eculizumab 1200 mg IV 1 h before kidney allograft reperfusion Eculizumab 900 mg IV post-trnx days 1,7, 14, 21, 28 Eculizumab 1200 mg post-trnx days 35, 49, 63^79^	116	Patients receiving eculizumab treatment (*N* = 52) showed a decreased 3-mo incidence of rejection (17%; 95% CI, 8 to 30) compared with that of patients receiving SOC (*N* = 64; 33%; 95% CI, 22 to 46; *P* = 0.06)
C1 esterase inhibitor (C1-INH) CSL Behring Inhibit activation of complement and intrinsic coagulation pathway	Vo *et al.*, 2015	NCT01134510	Pilot single arm, single center Phase 1/2	Prevention of AMR: 20 IU/kg IV at transplant, then twice weekly ×7 doses^88^	20	At 6 mo, no patients in the C1-INH group developed AMR versus 1 in placebo. C1-INH treatment significantly reduced C1q^+^HLA antibodies. In addition, post-transplant, C1-INH treated patients had lower DSA rebound rates and *dn*DSA development versus placebo
	Viglietti *et al.*, 2016	—	Pilot single arm, single center	AMR treatment: 20 units/kg on days 1, 2, and 3 and then twice weekly with IVIg 2 g/kg monthly for 6 mo^100^	6	All patients (*N* = 6) showed an improvement in eGFR, with mean eGFR of 38.7±17.9 and 45.2±21.3 ml/min per 1.73 m^2^ at baseline (0 mo) and 6 mo, respectively (*P* = 0.03)
BIVV009 Sanofi Humanized IgG4 Anti-c1s mAb	Eskandary *et al.*, 2018	NCT02502903	First-in-human, phase 1b, open label	Four weekly doses (60 mg/kg) for treatment of AMR^91^	10	BIVV009 blocked alloantibody triggered classical pathway complement activation, but did not impact characteristics of late AMR.
BIVV020 Sanofi Humanized IgG4 Anti-c1s mAb	Not yet published	NCT05156710	Phase 2, open-label RCT	Cohort A: evaluate the efficacy of BIVV020 in prevention of AMR Cohort B: evaluate the efficacy of BIVV020 in treatment of active AMR^101^	54	Study is ongoing (now enrolling)
**Anti-FcRn**						
Anti-FcRn (LALA mutated rhesus IgG1 chimeric of rozanolixizumab) Rozanolixizumab-noli. UCB, Brussels, Belgium Humanized recombinant high affinity monoclonal blocking FcRn	Manook *et al.*, 2021	—	Preclinical	Anti-FcRn LALA mutated rhesus IgG chimeric of rozanolixizumab 30 mg/kg anti-RhFcRn IV given to *n* = 6 monkeys. 60 mg/kg IV on day −5, 0 and +5 of transplant (*N* = 2)^71^	Six rhesus monkeys were treated	Anti-FcRn 30 mg/kg promoted reductions in total IgG and donor specific IgG. The 60 mg/kg dose did not lead to a greater reduction in IgG, of DSA IgG, which suggested saturation

ABMR, acute antibody mediated rejection; ABO, ABO blood group compatible; AMR, antibody-mediated rejection; APC, antigen presenting cell; BCMA, B-cell maturation antigen; BPAR, biopsy proven acute rejection; cAMR, chronic antibody mediated rejection; cABMR, chronic active antibody mediated rejection; CI, confidence interval; CMR, T-cell–mediated rejection; COVID, coronavirus disease; cPRA, calculated panel reactive antibody; CSA, cyclosporine; CTLA-4, cytotoxic T-lymphocyte antigen 4; CTLA4-Ig, cytotoxic T-lymphocyte antigen 4-Ig; DDKT, deceased donor kidney transplantation; DES, desensitization; dnDSA, *de novo* donor-specific HLA antibody; DSA, donor-specific HLA antibody; FDA, US Food and Drug Administration; FcRn, Fc neonatal receptor; GI, gastrointestinal; HLAi, HLA incompatible; HS, highly HLA sensitized; IL-6R, IL-6 receptor; IV, intravenous; IVIg, intravenous Ig; KT, kidney transplant; LALA, L234A/L235A; LDKT, living donor kidney transplantation; LTE, long-term extension; MAC, membrane attack complex; MFI, mean fluorescence intensity; NIH, National Institutes of Health; NS, normal saline; PC, plasma cell; PLEX, plasmapheresis; QM, every month; RCT, randomized controlled trial; RhFcRn, rhesus neonatal Fc receptor; SOC, standard of care; SQ, subcutaneous; TCZ, tocilizumab.

## Translating Advances in B-Cell Immunology into Effective Desensitization Therapies

Advances in B-cell immunology have been critical to understanding the antigenic anatomy of B cells, PCs, and effector elements (natural killer [NK] cells and macrophages) of antibody-mediated responses to allografts. The information gleaned from understanding the importance of anti-HLA antibodies and cellular elements that support their production and effector functions has allowed us to develop novel compositional approaches for the treatment of allosensitized patients. Critical to the first element, anti-HLA antibodies, newly developed agents such as imlifidase and Fc neonatal receptor (FcRn) inhibitors, and anti-CD38 antibodies address antibody-dependent cellular cytotoxicity (ADCC) and complement-dependent cytotoxicity (CDC) injury, which is the critical first step in a successful desensitization protocol.

**Figure 1:**
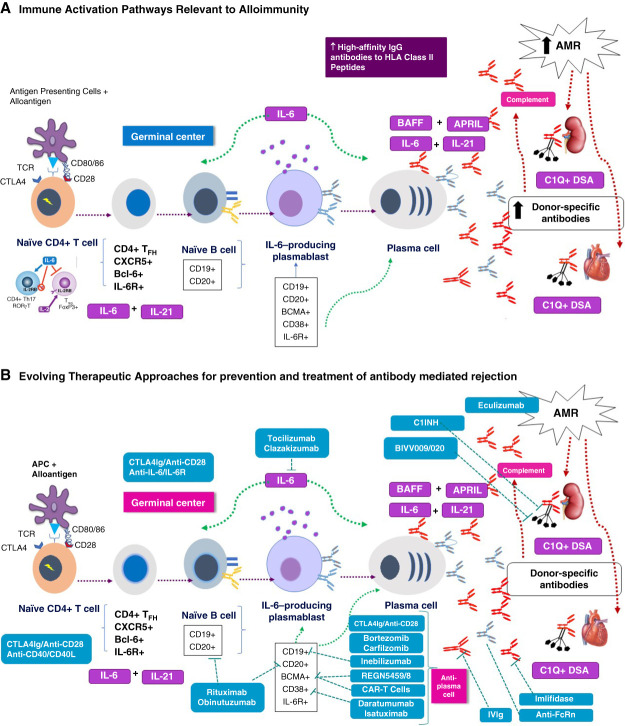
**Key cytokines for immune activation.** (A) The immune activation pathways critical for development of alloimmunity. APCs process and present alloantigens to naïve CD4^+^ T cells. This process requires alloantigen binding to cognate TCR complexes followed by costimulation through CD80/86 (APC) and CD28 (naïve T cell). Activated CD4^+^ cells then migrate to regional lymph nodes and spleen. Here, under the influence of CXCR5, they mature into T-follicular (T_FH_) cells that stimulate alloantigen responses in naïve B cells stimulated by the cytokines (IL-6 and IL-21). Activated B-cells develop into B_MEM_ cells with specific cells evolving into plasmablasts and ultimately to antibody-producing PCs. BAFF and APRIL are also critical to this process, interacting with BCMA (BCMA on PCs and some B cells). Antibody affinity to graft alloantigens (DSAs) evolve from low-affinity IgG (purple) to high affinity, complement activating alloantibodies (in red) binding to donor-specific HLA molecules in the allograft, and initiate the clinical and pathologic features of AMR. The antigenic anatomy of each cell type is shown that could represent relevant targets for therapeutic intervention. (B) The therapeutic approaches for prevention and treatment of AMR that are shown in conjunction with their targeted alloantigen activation and effector pathways. As discussed in the text, the major pathogenic factor is high-affinity IgG complement activating DSAs. Initial alloactivation events leading to T-cell activation can be blocked by CTLA4Ig or anti-CD28. Importantly, these costimulatory blockers are also potent inhibitors of germinal center (Tfh) activity and can prevent primary immune events induced by alloantigens. New data also suggest that CTLA4-Ig may directly inhibit CD80/86+ PCs. Inebilizumab (anti-CD19) depletes B cells and PB and approximately 50% of PC, anti–IL-6 (clazakizumab), or anti–IL-6R (TCZ) block activation of T_FH_ cell, Th17, and PB. Anti-PC therapies inhibit production of complement activating DSAs these include (daratumumab, isatuximab (anti-CD38), inebilizumab (anti-CD19), REGN5459 (bispecific anti-BCMA × CD3). Once pathogenic DSAs are present in copious amounts, imlifidase can cleave all IgG molecules, inhibiting CDC/ADCC. FcRn is an HLA class 1 molecule that is present in most cells and is responsible for recycling IgG molecules. Inhibition of FcRn with monoclonals and Fc fragments or saturation with IVIg enhances pathogenic IgG degradation, limiting pathogenesis. Complement inhibitors (C1-INH and anti-C5) can inactivate effector pathways critical to allograft injury (CDC). Combining antibody reduction therapies along with inhibition of B-cell activation and PC DSA production represents an achievable pathway for prevention and treatment of AMR. ADCC, antibody-dependent cellular cytotoxicity; AMR, antibody-mediated rejection; APC, antigen presenting cell; APRIL, a proliferation inducing ligand; BAFF, B-cell activating factor of TNF family; BCMA, B-cell maturation antigen; CDC, complement dependent cytotoxicity; CTLA4-Ig, cytotoxic T-lymphocyte antigen 4-Ig DSA, donor-specific HLA antibody; FcRn, Fc neonatal receptor; IL-6R, IL-6-receptor; IVIg, intravenous Ig; PB, plasma blasts; PC, plasma cells; TCR, T-cell receptor; TCZ, tocilizumab; T_FH_, T-follicular helper.

An ideal desensitization agent should reduce donor-specific HLA antibody (DSA), prevent CDC and ADCC, and most importantly, reduce deleterious antibody rebound. Antibody rebound is a well-recognized but poorly defined phenomenon characterized by rapid increases in pathogenic antibodies after removal by plasma exchange or IgG (by imlifidase). We assume that key cytokines, such as B-cell activating factor of TNF family/a proliferation inducing ligand and IL-6, stimulate B_MEM_ cells and PCs to rapidly produce antibody after these therapies. These pathways are summarized in (Figure 1A). In addition, therapeutics aimed at decreasing antibody levels and targeting DSA effector functions, CDC, and ADCC are essential^15^ (Figure 1B).

Current approaches to desensitization include plasmapheresis (PLEX) and intravenous Ig (IVIg). PLEX removes circulating antibodies (along with other plasma proteins). Although PLEX is generally considered a first-line therapy for desensitization, efficacy data are limited and are derived from small, single-center studies.^16–18^

Our group first reported on high-dose IVIg used for desensitization.^19^ IVIg, derived from pooled plasma of thousands of donors, has numerous effects on antibodies and effector cell functions, including blocking FcRn to limit antibody recycling, thus reducing antibody half-life, neutralizing pathogenic antibody effector functions *via* idiotypic or anti-idiotypic reactions, inhibiting complement activation, and activating immune inhibitory signaling through FcƴRIIb receptors on immune cells.^20^ IVIg is an important component of current desensitization protocols. It is also beneficial in preventing opportunistic infections in patients undergoing desensitization.^21^ However, the combination of IVIg+PLEX does not prevent the activation of well-established B_MEM_ and donor-reactive PCs producing pathogenic antibodies.

## Targeting Critical Effector Pathways ADCC and CDC

ADCC and CDC are critical effector pathways mediating pathogenic antibody injury and destruction of cognate antigen-binding targets. The ability of IgG Fc receptors to recognize antibodies bound to these targets is essential to this effector function. In kidney transplantation, the targets are usually HLA antigens on donor vascular endothelium. For CDC, critical segments of the Fc region of DSAs activate C1Q and initiate the classic complement pathway to form C5b–C9 membrane attack complex (MAC), which destroys endothelial cells and results in graft injury. ADCC is mediated by NK cells, monocytes, and polymorphonuclear leukocytes. Fc-receptor binding initiates cytotoxic enzyme release (perforin and granzyme), destroying the endothelium and perpetuating graft injury. Both processes are important and can occur in concert (or separately). However, it appears that in early severe graft rejection, CDC is more critical, whereas in chronic antibody-mediated rejection (cAMR), ADCC plays a more important role.

Anti–B-cell therapy for desensitization and AMR treatment was recognized with the known association between B-cell responses in AMR and reduced allograft survival.^22–25^ Our desensitization protocols include anti-CD20 (rituximab or biosimilar equivalents) as an essential component, depleting B cells and likely preventing primary sensitization and recall antibody responses by memory B-cell depletion.^22–24,26–28^ Comprehensive reviews of desensitization using IVIg and anti-CD20 agents have been published.^13,14,29–31^ Anti-CD20 therapeutic advancement, including obinutuzumab (type 2 anti-CD20) development, demonstrated clinical superiority to rituximab in B-cell lymphoma and systemic lupus erythematosus treatment.^32^ IVIg+obinutuzumab for desensitization failed to demonstrate efficacy in decreasing anti-HLA antibodies in HS patients.^33^ However, obinutuzumab demonstrated significant reductions of B cells, B_MEM,_ and plasmablasts in patients with ESKD. This suggests that obinutuzumab may be important in modifying rebound antibody responses from B_MEM_ cells and plasmablasts. It is important to note that obinutuzumab is very effective in reducing/eliminating autoantibodies versus alloantibodies. This likely represents a targeting issue because autoantibodies are primarily generated from CD20^+^ plasmablasts while HLA antibodies are produced by long-lived PCs, which are CD20 deficient.

## IL-6/IL-6 Receptor Inhibition

IL-6 is a multifunctional cytokine critical for T-follicular/helper cells (T_fh_) and adaptive immune responses.^34,35^ A review of data investigating inhibition of IL-6/IL-6 receptor (IL-6R) signaling pathway suggests that it may provide benefits in reducing pathogenic IgG antibodies and T-effector/memory responses while increasing T_REG_ populations, limiting endothelial cell activation and injury in response to DSA binding.^36^

### Tocilizumab (Anti–IL-6R)

Tocilizumab (TCZ) is a humanized anti–IL-6R mAb. Our group evaluated TCZ+IVIg desensitization in ten HS kidney transplant (KT) recipients who failed standard desensitization.^37^ Five patients were transplanted. No AMR was seen on 6-month protocol biopsies, and DSA reductions occurred. Daligault *et al.* reported on TCZ administration to treatment-naïve HS patients (*N* = 14).^38^ TCZ significantly reduced dominant anti-HLA antibody sensitization. Mean fluorescence intensity (MFI) decrease was minor, with only one patient transplanted. Jouve *et al.* conducted the TETRA study where monthly TCZ × 6 months, given before standard of care regimen, found TCZ+standard of care could limit post-transplant HLA antibody rebound, but reductions in pretransplant MFIs were not clinically significant.^39^

### Clazakizumab (Anti–IL-6)

Clazakizumab, an IgG1 anti–IL-6 mAb,^40^ was evaluated for desensitization at our center. Here, 20 HS patients (cPRA > 50%) received PLEX + IVIg and then clazakizumab monthly × 6 months.^41^ If transplanted, patients received clazakizumab × 12 months. After 6 months, clazakizumab treatment was associated with significant reductions in HLA antibodies, including strong-binding HLA antibodies (>10,000 MFI) with 18 patients transplanted. Fourteen patients (78%) were DSA positive at transplant. At 12 months, only one patient remained DSA positive, patient and graft survival were 100% and 94%, respectively, and the mean eGFR was 58 ± 29 ml/min per 1.73 m^2^. Two other clazakizumab studies in patients with cAMR^42,43^ showed reduced DSA levels, pathologic and molecular features of cAMR, and, importantly, eGFR stabilization. However, preliminary data analysis from a phase 3 randomized controlled trial of clazakizumab for cAMR treatment in patients undergoing KT (IMAGINE) failed to show efficacy at 1 year.^44^ This study is now closed. Nonetheless, anti–IL-6/IL-6R treatments are likely important in desensitization and DSA rebound prevention and still hold promise for treatment of cAMR in certain populations.

## PC-Directed Therapies

Because long-lived PCs are the key producers of HLA antibodies, it seems reasonable to develop PC-directed therapies.

## Proteosome Inhibitors

### Bortezomib

Bortezomib, a proteosome inhibitor used to treat multiple myeloma (MM), was examined for AMR treatment. Unfortunately, data from a well-conducted, placebo-controlled trial showed no significant capability to reduce DSAs, stabilize kidney function, or affect patient/graft survival.^45^ Desensitization studies showed no meaningful HLA antibody level declines.^46^ In addition, studies showed substantial adverse event/serious adverse events with bortezomib administration.

### Carfilzomib

Carfilzomib, a second-generation proteosome inhibitor, may be more robust in depleting PCs with less neuropathic side effects. Carfilzomib + PLEX were assessed for desensitization in 13 HS candidates undergoing KT.^47^ MFI was modestly reduced in most, but antibodies rebounded to baseline levels within 5 months. A study of carfilzomib in patients undergoing lung transplant with AMR appeared to decrease DSAs and improve lung function.^48^ One-year graft survival for patients responsive to carfilzomib treatment was 78% versus 20% for nonresponders. Another study in lung transplant recipients showed similar results.^49^ No deaths occurred during treatment, but at 1 year, seven of 14 patients had died from graft failure. Although promising, long-term therapy with agents preventing DSA rebound and allograft injury are likely required to sustain initial benefits seen with carfilzomib.

## Monoclonal Antibodies Directed at PCs

### Daratumumab

Daratumumab is a humanized IgG1k mAb with specificity for CD38 transmembrane glycoproteins on PCs, plasmablast, NK, and T_REG_ cell surfaces.^50^ In a primate model of allosensitization, daratumumab reduced DSAs and slightly improved kidney allograft survival,^51^ but a rapid antibody rebound with intense T cell–mediated rejection (CMR) was seen at 1 month, suggesting short-lived PC depletion. Jordan *et al.* reported using daratumumab for desensitization in a HS heart transplant candidate and resistant AMR treatment in a KT recipient.^52^ HLA antibodies decreased in both patients. AMR findings improved in the patients undergoing KT after daratumumab, but CMR developed on subsequent biopsy. This was also reported by others.^53,54^ Vo *et al.* reported daratumumab use for desensitization in ten HS patients, who failed several previous desensitization treatments before receiving daratumumab ± PLEX treatment.^55^ Daratumumab reduced DSA MFI strength and enabled HLA incompatible transplantation in eight of ten patients. At 12 months, patient/graft survival was 100%/100% and eGFR was 73±22 ml/min per 1.73 m^2^. Daratumumab was well tolerated without significant SAEs.

Although promising, daratumumab exhibits numerous off-target immune effects (*i.e*., CD38^+^ T_REG_ and B_REG_ cell depletion) likely increasing CMR risk.^56^ The ability to deplete NK cells and monocytes may account for improvements in glomerulitis and PTCitis observed after daratumumab treatment of AMR.

### Isatuximab

Isatuximab is an anti-CD38 approved for treatment of MM. Vincenti *et al.* reported on a clinical trial of isatuximab for desensitization. Overall, cPRA values were minimally affected, with only 9 of 23 patients (39%) having cPRA reductions to target levels. By study cutoff, six patients received transplant offers, of which four were accepted. Isatuximab was considered safe with some effect on PRA reduction.

## Costimulatory Blockade and Combination Therapy

Costimulatory blockade primarily inhibits naïve CD4^+^ T-cell activation by impeding CD28/B7-1/B7-2 costimulation.^57,58^ The prototype fusion protein (cytotoxic T-lymphocyte antigen 4-Ig [CTLA4-Ig]) interferes with signal 2. Data from in vitro models suggest that naïve B cells activated by T_FH_ cells in germinal centers are inhibited by CTLA4-Ig.^58^ This likely explains the benefits of CTLA4-Ig on limiting *de novo* DSA development. CTLA4-Ig inhibits IgG production by PCs in animal models but is not validated in humans.^58^ Importantly, CTLA4-Ig does not inhibit CD8^+^ T-effector/T-memory which contributes to the high rejection rates seen in patients receiving CTLA4-Ig without calcineurin inhibitor immediately post-transplant. Recent studies in a primate model of KT demonstrated the inability of CTLA4-Ig to modify CD8^+^ T-effector/memory cells in animals maintained on CTLA4-Ig alone. However, this was ameliorated with TCZ coadministration^59^ with significant reductions in CD8^+^ cell populations.

Chandran *et al.* reported preliminary data from a phase 1/2 trial evaluating daratumumab followed by belatacept as desensitization in cPRA ≥99.9% patients,^60^ with one patient transplanted. Jackson *et al.* also reported a pilot study of carfilzomib with belatacept for desensitization in HS patients.^61^ Two patients met the efficacy end point (eliminating one HLA antibody or ≥50% MFI reduction of ≥3 HLA antibodies). Although encouraging, more data are needed.^61^

Jain *et al.* assessed the efficacy of bortezomib+CTLA4-Ig in six patients undergoing KT as rescue therapy for acute AMR or mixed rejection.^62^ DSA (class 1 and 2) reductions were seen within 25 days of treatment. DSA levels remained low or undetectable for a 10- to 30-month follow-up.

## Imlifidase

Imlifidase, or IdeS (IgG-degrading enzyme derived from *Streptococcus pyogenes*), is a novel IgG endopeptidase showing promise for desensitizing HS patients.^63–65^ Imlifidase cleaves IgG antibodies at the IgG hinge region, resulting in two fragments, F(ab')2 and Fc. This enzymatic separation of the intact IgG components disrupts IgG-mediated ADCC and CDC. Imlifidase cleaves the B-cell receptor from B cells and selectively cleaves IgG antibodies without affecting other immunoglobulin molecules. This allows the beneficial immune responses rendered by other antibody types to remain.

Desensitization studies using imlifidase are promising. In a phase 1/2 trial, imlifidase given to HS patients before transplantation (*N* = 25) completely eliminated HLA antibodies with 24 patients transplanted.^65^ Data from this and other studies resulted in conditional approval of imlifidase by the European Medicines Agency for desensitization of cross-match positive individuals awaiting deceased donor kidney transplantation.^66^ Imlifidase is being evaluated in a phase 3 trial (ConfIdeS) for desensitization in HS (>99.90%) patients undergoing KT (NCT05369975).^67^

Significant issues limit imlifidase use. Importantly, it can only be used once because of immunogenicity resulting in anti-imlifidase neutralizing antibody formation^68^; this has encouraged attempts to develop a less immunogenic imlifidase. In addition, imlifidase needs to be accompanied by other treatments to reduce rebound IgG production.

## FcRn Inhibitors

Another potential approach to desensitization uses novel agents to inhibit FcRn recycling, which decreases pathogenic IgG half-life. Here, identification of FcRn recycling of IgG to extend IgG half-life (approximately 28 days) was an important breakthrough. Notably in FcRn^(−/−)^ animals, IgG half-life is only 3 days. In depth reviews of FcRn potential applications in transplantation are available.^69,70^ Knechtle *et al.* assessed the efficacy of anti-FcRn monoclonals administered before and after kidney transplantation in rhesus macaque monkeys, sensitized by skin grafting.^71^ The authors found perioperative anti-FcRn mAb administration interfered with total circulating IgG *via* FcRn-mediated recycling, without reducing levels, likely because of ongoing DSA produced by PCs. The authors concluded that anti-FcRn treatment transiently reduced DSAs without affecting DSA synthesis.

**Figure 2:**
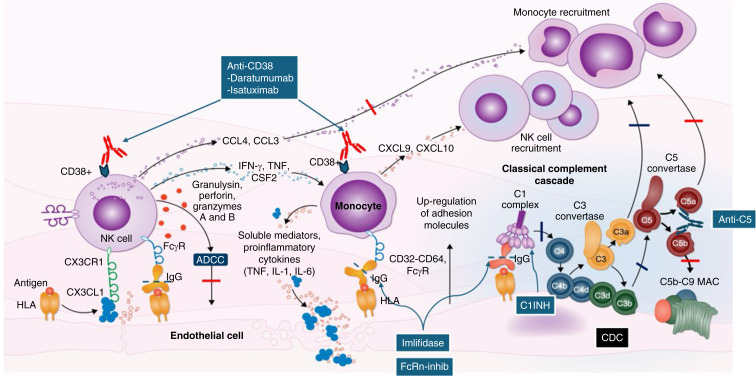
**This figure demonstrates the importance of ADCC and CDC as critical antibody effector pathways responsible for endothelial cell injury and ultimately, antibody rejection.** First, ADCC is mediated by NK cells, monocytes, and PMNs through recognition of Fc fragments of pathogenic DSAs bound to endothelial cell targets (HLA molecules). Interaction of FcƴR+ cells with Fc of target bound DSAs initiates cell activation with the release of perforins and granzyme, destroying the target cell. NK cells also release chemokines and cytokines that result in recruitment of monocytes and macrophages that increase ADCC and cell adhesion to the target endothelium. CDC is mediated primarily by activation of the classic complement pathway by pathogenic DSAs. This activation ultimately results in formation of the C5b–C9 MAC which destroys target cells. During complement activation, potent anaphylatoxins (C3a and C5a) are released and are critical for initiation of monocyte and NK cell recruitment. In addition (not shown) C3a and C5a bind to receptors on B cells and T cells resulting in activation of T-cell–directed immune responses and enhancement of high-affinity DSA production. Important therapeutic agents to combat ADCC and CDC include imlifidase which removes Fc from DSAs and thus inhibits their ability to mediate these important injurious pathways. This also likely true for the new FcRn inhibitors which rapidly reduce circulating IgG (DSA) by inhibiting IgG recycling mechanisms. This would also be true for high dose IVIg. In addition, anticomplement therapies (anti-C5 and C1-INH) inhibit classic, mannose binding and alternative pathways, limiting CDC and activation of immune cells. ADCC can also be inhibited by use of anti-CD38 which can deplete NK cells and monocytes/macrophages as shown. (Modified from: Peerview.com/RBD.) MAC, membrane attack complex; NK, natural killer; PMN, polymorphonuclear leukocyte.

The first US Food and Drug Administration–approved FcRn inhibitor, efgartigimod, is an Fc fragment with high affinity for the FcRn, resulting in reduced circulating IgG half-life. Efgartigimod was approved for the treatment of myasthenia gravis.^72^ Clinical trials are being planned for treatment of AMR. Figure 2 summarizes the effector pathways of CDC and ADCC and their importance in mediating injury to allograft endothelial cells. This demonstrates how therapies aimed at inhibiting CDC/ADCC are critical to prevent graft endothelium injury.^73^

## Bispecific Antibody Targeting B-Cell Maturation Antigen and CD3

REGN5459/5458 are anti–B-cell maturation antigen (BCMA)×anti-CD3 bispecific antibodies, binding BCMA on PCs and CD3 on cytotoxic T lymphocytes. REGN5459/5458 brings CD3^+^ cells in proximity to PCs with subsequent PC killing by the activated CD3^+^ cell. A phase 1/2, open-label study of REGN5459 (or REGN5458) to desensitize HS patients with ESKD is ongoing.^74^ REGN5459/5458 may offer benefits over anti-CD38 which targets multiple cell types because BCMA is primarily located on PCs. REGN5459/5458 appears to be effective in early MM clinical trials.^75^ Here, it is important to discern the antigenic anatomy of PCs responsible for HLA DSA generation. If they specifically express BCMA, this approach could be promising.

## Chimeric Antigen Receptor-T Cells (CD19) and Chimeric Antigen Receptor-T Cells (BCMA)

Studies are underway to examine chimeric antigen receptor (CAR) T cells (CAR-T; CD19 and BCMA) as potential desensitization agents (NCT06056102). Currently, there is little information on this approach; however, Jackson *et al.* reported a retrospective analysis of HLA antibodies in HS patients receiving CAR-T (CD19) treatment for MM.^76^ Interestingly, the investigators saw no meaningful reductions in HLA antibodies after CAR-T (CD19) treatment. CAR-T (BCMA) impact on HLA antibodies was not analyzed. Investigators concluded that HLA antibody producing cells are likely CD19^NEG^. CD19 is expressed on all B cells and approximately 50% of PCs. This may be likely the experience with obinutuzumab (anti-CD20), which depletes plasmablasts and some PCs, without affecting HLA antibodies in HS patients.^32,77^ It is important to determine whether there are meaningful differences in efficacy of CAR-T cells compared with bispecific antibodies which mimic CAR-T cells actions.

## Complement Inhibition

### Eculizumab (Anti-C5)

Eculizumab is a mAb aimed at complement activation initiated by C5 convertase resulting in formation of the MAC (C5b-C9MAC). Treatment results in terminal complement inhibitions by preventing (C5b-C9MAC) formation. Stegall *et al.* showed that eculizumab significantly reduced AMR incidence compared with a historical control of patients desensitized with PLEX + IVIg (7.7% (2/26) versus 41.2% (21/51; *P* = 0.003).^78^ Although the results were encouraging, patients receiving long-term eculizumab with DSA positivity did not show a difference in cAMR rates versus controls. This is an important demonstration of the need for antibody reduction therapies combined with CDC inhibition for good outcomes. Without DSA depletion, ADCC is not mitigated and results in cAMR.

Lefaucheur *et al.* reported on 931 KT recipients who had determinations of complement activating DSAs, biopsy assessments, and molecular scores.^79^ The investigators reported that eculizumab significantly improved graft survival and was specifically effective against rejection phenotypes associated with complement activating DSAs. Assessment of C1Q activating DSAs is not done in most US centers. Here, one can usually assume that DSAs of >10,000 MFI are complement activating.^80^

Importantly, eculizumab use for desensitization needs careful consideration. First, it increases expense. Second, eculizumab has a very short half-life because it is not Fc-modified to enhance recycling through the FcRn system. There are novel strategies to improve the half-life of eculizumab through Fc engineering to enhance Fc/FcRn interactions at pH 6.5 and demonstrate an improved antibody half-life and therapeutic efficacy.^81,82^ Ravulizumab, an Fc-engineered anti-C5, developed from eculizumab, has enhanced binding to the FcRn. IgG-Fc/FcRn interactions prolong therapeutic antibody half-life. In addition, alterations in F(ab) amino acid structure allows C5 release at pH 6.5 when anti-C5 is bound to FcRn, allowing ligand (C5) degradation in endosomes. This allows refreshed anti-C5 to be released into the circulation, allowing continuous C5 shuttling to the endosomes for degradation. In summary, anti-C5 remains a valuable therapy for AMR where clear evidence of rapid graft decline is associated with complement (C4d) deposition and the presence of complement-activating anti-HLA antibodies.

### C1 Esterase Inhibitor (C1-INH)

C1-INH prevents complement activation *via* the classical and MBL pathways. C1-INH has been investigated as an adjunct to desensitization. Data from animal models^83,84^ and a human trial by Jordan *et al.* suggest that C1-INH treatment ameliorates ischemia/reperfusion injury.^85,86^ Here, C1-INH's prevention of ischemia/reperfusion injury likely prevents immune activation events within the allograft, leading to B-cell activation and alloantibody production.^87^ Vo *et al.* also investigated C1-INH for AMR prevention in HS KT recipients, with acceptable safety outcomes.^88^ C1-INH treatment significantly reduced C1q^+^ HLA antibodies. In addition, post-transplant, C1-INH treated patients had lower DSA rebound rates and *de novo* DSA development versus placebo. This is consistent with the C1q-initiated complement activation that drives antibody affinity maturation.^89^

In a study evaluating a humanized mAb targeting C1s, investigators showed inhibition of alloantibody initiated classical pathway complement activation *in vitro*.^90^ The authors performed a trial of anti-C1s in AMR patients (NCT02502903).^91^ There were no severe AEs, and anti-C1s completely eliminated DSA-triggered classic pathway activation. In addition, C4d^+^ staining was significantly reduced in most patients. Despite this, there were no meaningful reductions in microcirculation inflammation, gene expression patterns, DSA levels, or kidney function improvement. Again, selective CDC inhibition, without DSA reductions, has limited benefits (Table 1).

## Conclusions

Currently, there is a rapid emergence of new agents aimed at desensitization and AMR treatment. Multiple agents with varying mechanisms of action include imlifidase and FcRn inhibitors, anti-cytokine (IL-6/IL-6R) therapies, and PC-directed therapies. However, no single agent can control all facets of antibody generation and injury. The future of antibody-directed therapeutics will require protocols consisting of several agents placed in a logical sequence to rapidly remove pathogenic DSAs and prevent their reemergence.
